# Combined hyperthermia and chlorophyll-based photodynamic therapy: tumour growth and metabolic microenvironment

**DOI:** 10.1038/sj.bjc.6601457

**Published:** 2003-12-09

**Authors:** D K Kelleher, O Thews, A Scherz, Y Salomon, P Vaupel

**Affiliations:** 1 Institute of Physiology and Pathophysiology, Johannes Gutenberg-University Mainz, Duesbergweg 6 D-55099 Mainz, Germany; 2 Department of Plant Science, The Weizmann Institute of Science, 76100 Rehovot, Israel; 3 Department of Biological Regulation, The Weizmann Institute of Science, 76100 Rehovot, Israel

**Keywords:** photodynamic therapy, bacteriochlorophyll, hyperthermia, blood flow, oxygenation, metabolic status

## Abstract

The effects of combined and simultaneously applied localised 43°C hyperthermia (HT) and an antivascular bacteriochlorophyll-serine-based photodynamic therapy (Bchl-ser-PDT) on tumour growth and several microenvironmental parameters were examined. Rats bearing DS-sarcomas were allocated to treatment groups: (i) sham-treatment (control), (ii) Bchl-ser-PDT (20 mg kg^−1^ i.v.), (iii) localised HT, (iv) Bchl-ser-PDT+HT. The light source used was an infrared-A irradiator, which, by use of appropriate filters, delivered the different ranges of wavelengths required. Following treatment, tumour volume was monitored. The greatest tumour growth inhibition was seen with Bchl-ser-PDT+HT, and subsequent experiments identified the pathophysiological basis for this effect. Red blood cell flux in tumour microvessels declined rapidly upon Bchl-ser-PDT+HT, reaching approximately 10% of initial values by the end of treatment. Similarly, tumour oxygenation worsened, reaching almost anoxic levels by the end of the treatment period. Assessment of metabolic parameters showed a pronounced increase in lactate levels and a decrease in ATP concentrations after combined treatment. The results presented suggest that vascular collapse and flow stasis resulting in a deterioration of tumour oxygenation and a switch from oxidative to glycolytic glucose turnover are key elements in the tumour eradication seen with this novel approach in which an antivascular PDT and HT are combined and simultaneously applied.

Photodynamic therapy (PDT) is increasingly becoming accepted as a treatment option for a variety of oncological, dermatological, ophthalmic and cardiovascular diseases (Dougherty, 2002). In oncological applications, treatment is usually based on the photosensitisation of tumour cells with subsequent light exposure leading to death of the malignant cells. The most commonly used photosensitisers have been the haematoporphyrin derivatives, which do however have a number of drawbacks including an often poor selectivity in terms of tumour drug accumulation and low extinction coefficients so that relatively large amounts of drug and/or light are needed in order to obtain a satisfactory phototherapeutic response. Additionally, light of wavelengths corresponding to the absorption maximum at approximately 630 nm only penetrates poorly into tissues, thus presenting a limitation concerning the depth of treatment (usually less than 4–5 mm). Due to a high drug accumulation in normal skin, patients may also be confronted with skin phototoxicity which may persist for up to 8 weeks after treatment. These issues have led to the development of a further generation of photosensitisers, a number of which are already being investigated for clinical application. One potentially interesting group of substances are the chlorophyll derivatives. Of these, bacteriochlorophyll-serine (Bchl-ser) – the photosensitizer used in the present study – shows a number of features that underline the potential of soluble bacteriochlorophyll derivatives in tumour photodynamic therapy. These features include phototoxicity, which under *in vitro* conditions is approximately 200 times greater than the haematoporphyrin derivatives, strong absorption at 760–80 nm (allowing deeper light penetration into tissues), rapid tissue clearance and minimal extravasation from the circulation (Rosenbach-Belkin *et al*, 1996).

Traditionally in PDT – as with chemo- and radiotherapy - the primary treatment target was the tumour cell. Although vascular injury was considered to be important in the process of tumor eradication, it was feared that partial vascular shutdown, as observed with some photosensitisers, may limit treatment efficacy due to a reduction in blood flow and a subsequent decrease in O_2_ delivery (Henderson and Fingar, 1989). More recently, however, attention has been given to the possibility of treating solid tumours by PDT targeted at the tumour vasculature (Fingar, 1996,^1999^; Chen *et al*, 2002). Certainly, when PDT is applied for age-related macular degeneration, the blood vessels are the sole treatment target (Dougherty, 2002). In animal experiments, Bchl-ser was found to produce maximum antitumour effects when illumination was applied close to the time of drug injection when the photosensitiser concentration in blood was at its highest (Zilberstein *et al*, 2001), with microscopic examination primarily revealing extensive vascular damage. Bchl-ser thus, besides exhibiting a number of favourable photosensitiser properties, appears to be a suitable candidate for use in PDT targeted at the tumour vasculature.

The aim of the present study was to attempt a novel combination of an antivascular PDT treatment and localised hyperthermia (HT) for enhanced antitumour therapy. Clinically, HT alone is not an appropriate option for the curative treatment of human tumours. However, a number of clinical studies have indicated a clear benefit when HT was combined with other therapy modalities, in particular radiotherapy (Overgaard *et al*, 1996; Vernon *et al*, 1996; Prosnitz *et al*, 1999; van der Zee *et al*, 2000). As far as a combination of HT with PDT is concerned, a number of *in vitro* and *in vivo* studies suggest that the antitumour effect of PDT may be enhanced by HT (Christensen *et al*, 1984; Henderson *et al*, 1985; Mang and Dougherty, 1985; Levendag *et al*, 1988; Chen *et al*, 1996; Rasch *et al*, 1996; Kelleher *et al*, 1999; Orenstein *et al*, 1999). Considering the results obtained in such studies, a simultaneous delivery of light and heat, though technically more challenging, may be preferable in terms of a maximum cytotoxic effect, and certainly, from a clinical point of view, a therapy combination which can be applied in a single session would be favourable. A method for the application of PDT and controlled localised hyperthermia in a single session has previously been described by our group (Kelleher *et al*, 1999), and the present paper attempts to examine the antitumour benefit which might be achieved by applying the combination of a vascular-targeted PDT treatment simultaneously with HT. At the same time, the consequences of such a therapy for the tumour microenvironment, in terms of perfusion, oxygenation and metabolic parameters, have been examined for the first time using an experimental tumour model in the rat, since modifications of these parameters can crucially impact on the therapeutic outcome.

## MATERIALS AND METHODS

### Animals and tumours

Solid tumours grew subcutaneously following injection of DS-sarcoma cells (0.4 ml approx. 10^4^ cells *μ*l^−1^) into the hind foot dorsum of male Sprague–Dawley rats (Charles River Wiga, Sulzfeld, Germany; receiving a standard diet and water *ad libitum*; body weight at time of tumour implantation: 180–210 g). Approx. 1 week after implantation, when a tumour volume of 0.7–1.0 ml had been reached, animals were anaesthetised with sodium pentobarbital (40 mg kg^−1^ i.p., Narcoren®, Merial, Hallbergmoos, Germany). Throughout all experiments, animals lay supine on a thermostatically controlled heating pad and the rectal temperature maintained at 37.5–38.5°C. Animals breathed room air spontaneously. In the case of the acute experiments (laser Doppler flux, oxygenation status, metabolite concentrations), polyethylene catheters were placed into the thoracic aorta via the left common carotid artery and into the right external jugular vein (to allow administration of additional anaesthetic, as necessary). Continuous monitoring of the mean arterial blood pressure (MABP) was possible via attachment of the arterial catheter to a pressure transducer (P23 ID, Gould, Oxnard, CA, USA). After the surgical procedures, animals were allowed to stabilise and treatment commenced once constant baseline readings for laser Doppler flux or oxygenation were obtained for at least 20 min.

### Photosensitiser

**Figure 1:**
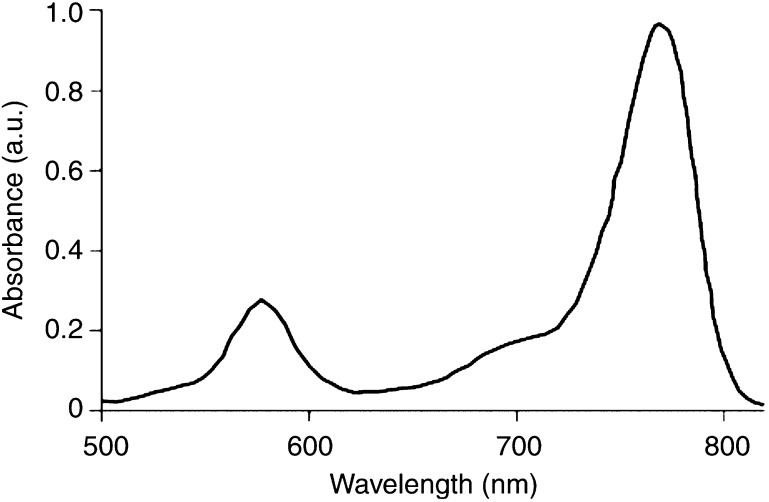
Absorption spectrum for a solution of bacteriochlorophyll-serine in acetone.

The photosensitiser used in this study was Bchl-ser. This water-soluble derivative of the natural pigment bacteriochlorophyll-*a*, was prepared as described previously (Scherz *et al*, 1994). The absorption spectrum for Bchl-ser is shown in Figure 1

. Subsequent to preparation, the photosensitizer was stored under argon at −20°C. The Bchl-ser was dissolved in 95% ethanol (5 mg/100 *μ*l) and then diluted in 1 ml phosphate-buffered saline immediately prior to administration via a tail vein so that animals received a dose of 20 mg kg^−1^ body weight. In the case of combined Bchl-ser-PDT+HT, the photosensitizer was injected 10 min following commencement of HT. Correspondingly, Bchl-ser-PDT involved photosensitizer injection 10 min after commencement of illumination. Control animals received an equivalent volume of ethanol (400 *μ*l kg^−1^) in 1 ml phosphate-buffered saline.

### Treatments

Group I (control): sham treatment. Group II (Bchl-ser-PDT): animals received Bchl-ser (20 mg kg^−1^ i.v.) and ‘non-thermal’ illumination (665–800 nm; 125 mW; 230 J cm^−2^). Group III (HT): localised HT at 43°C (665–1400 nm; 760 mW cm^−2^; 1596 J cm^−2^). Group IV (Bchl-ser-PDT+HT): animals treated with Bchl-ser (20 mg kg^−1^ i.v.) and localised tumour HT for 60 min at 43°C (665–1400 nm; 760 mW cm^−2^; 1596 cm^−2^; for this group, the light dose in terms of the effective spectral range of the photosensitising drug used in this study–from 665 to 800 nm was identical to that of group II, i.e., 230 J cm^−2^).

The radiation delivery system used has been described in detail previously (Vaupel *et al*, 1992; Kelleher *et al*, 1995,^1999^). The energy source is a halogen lamp emitting over the spectral range 420–1400 nm. As described above, experimental groups were treated with different wavelength windows. For group II (Bchl-ser-PDT), a long-wave pass (665 nm) and a short-wave-pass filter (800 nm) were inserted into the radiation path to obtain wavelengths 665–800 nm, so that the infrared bands which would otherwise result in heating were removed. Only the long-wave-pass filter was inserted in the case of groups III and IV (665–1400 nm) so that HT could be applied. The radiation system additionally encompasses a water filter responsible for two strong, distinct absorption bands at 944 and 1180 nm, which would otherwise be absorbed by the most superficial skin layers resulting in painful sensations and exsiccosis. The heating patterns achieved with this system, showing therapeutically relevant temperatures in tissues up to a depth of 1.2 cm, have been described (Vaupel *et al*, 1992; Kelleher *et al*, 1995), as have the resulting tumour temperature distributions (Kelleher *et al*, 1995). In groups where HT was applied (III and IV), a feedback control system involving the measurement of temperature in the tumour centre with a thermocouple (∅ 250 *μ*m; type 2ABAc, Philips, Kassel, Germany) was used to heat tumours (0.4°C min^−1^) to a set temperature of 43°C. Following the 20 min heat-up phase, the tumour temperature was maintained at 43°C for 60 min by regulation of the source which was switched on and off intermittently by the feedback control system, resulting in an average total energy dose of 1596 J cm^−2^ over the complete 80 min treatment period, as measured in pilot studies. These pilot studies were also used to obtain information concerning the pattern of irradiation and showed that, on average, the tumours used were irradiated for 1.3 s followed by a non-illumination period of 2 s over the 80 min treatment period. This pattern was subsequently used for the Bchl-ser-PDT treatment (Group II) so that the light delivered in the range from 665–800 nm was equal for all groups in which tumours were illuminated. The fluence rate for this wavelength range was 125 mW cm^−2^ and the total energy dose was 263 J cm^−2^. However, since the photosensitizer was administered 10 min after commencement of illumination (in order to correspond to the Bchl-ser-PDT+HT group), the ‘effective’ total energy dose (i.e., light involved in a photodynamic effect) was 230 J cm^−2^. A radiometer/photometer (IL1400A, International Light, Newburyport, MA, USA) with a calibration traceable to the National Physical Laboratory (USA) was used for light dosimetry purposes. The irradiation was applied solely to the tumour, the remainder of the animal being shielded by aluminium foil.

It should be noted that the light doses administered in the Bchl-ser-PDT group were not selected on the basis of conventionally used light doses in PDT, but rather arose from the properties of the light source used and the energy doses necessary to induce 43°C HT in those groups where tissue heating was induced.

### Assessment of *in vivo* tumour response

The three orthogonal diameters (*d*) of the tumours were measured on a daily basis so that tumour volume (*V*) could be determined using the ellipsoid approximation *V*=*π*/6 × d_1_ × d_2_ × d_3_. The end point of the tumour response study was a tumour volume of 3.5 ml, rather than survival. This target value was selected to ensure that the tumour burden at the end of the study never exceeded 1% of body weight. Animals whose tumours did not reach the target volume were monitored for 90 days.

### Laser Doppler flowmetry

Determination of the red blood cell (RBC) flux in tumour microvessels was performed using a laser Doppler flowmeter (Periflux 2B, Perimed, Stockholm, Sweden). This method relies on the Doppler shift, assessing the frequency change undergone by light when reflected by moving objects, such as RBCs, and is a valid technique for measurement of microcirculatory function in small tissue areas (Smits *et al*, 1986). RBC flux signals were obtained from a central location within the tumour using a needle probe (PF 302, o.d.: 0.45 mm). Using a 24-gauge needle, a small incision of the skin covering the tumour was made to allow insertion of the laser Doppler probe. Recording of the total backscattered light during the monitoring period ensured an optimum and constant probe positioning and minimum tissue compression. At the end of each experiment, the laser Doppler probe was left in place and the animal given an overdose of anaesthetic so that the biological zero RBC flux signal could be established and subtracted from all previous values for each animal. Data were subsequently expressed relative to the RBC flux obtained immediately prior to commencement of treatment.

### Tumour oxygen tension measurements

Polarographic assessment of tumour oxygenation was carried out using a flexible O_2_-sensitive catheter electrode (∅ 350 *μ*m, LICOX pO_2_, GMS, Kiel, Germany), which was inserted into the tumour for continuous monitoring. Calibration of the pO_2_ electrode was carried out using room air in a constant temperature chamber, taking into account the prevalent barometric pressure. Data were subsequently expressed relative to the value obtained immediately prior to commencement of treatment.

### Determination of metabolite concentrations

In a further series of experiments, the tumour-bearing foot of the anaesthetised animals was rapidly frozen in liquid N_2_ immediately after completion of treatment. Two-thirds of the tumour mass was then ground to a fine powder and freeze-dried. Glucose and lactate concentrations were then determined using standard enzymatic assay kits (Boehringer-Mannheim, Mannheim, Germany), and global ATP, ADP and AMP concentrations were measured with reversed-phase HPLC, as described previously (Krüger *et al*, 1991).

Cryosections (20 *μ*m) were prepared from the remainder of the tumour. These were heat-inactivated at 80°C for 5 min before storage at −80°C. Microregional distributions of lactate and ATP concentrations were subsequently assessed using single-photon imaging and bioluminescence techniques (for more details see Mueller-Klieser and Walenta, 1993). For this, a frozen tumour section mounted onto the underside of a glass microscope slide was placed onto another slide containing a well into which an aliquot of a cooled mixture of enzymes and coenzymes had been pipetted. Specific to the substrate undergoing investigation, these enzymes provide a link to a luciferase reaction. The whole preparation was warmed to 20°C and the spatial distribution of the bioluminescence intensity within the tissue section directly measured using a microscope (Axiophot, Zeiss, Göttingen, Germany) and an imaging photon-counting system (Argus 100, Hamamatsu, Herrsching, Germany). Using this method, a spatial resolution of approximately 50 *μ*m could be achieved. Heat-inactivated tissue homogenates with defined concentrations of lactate and ATP were treated in the same way as tumour sections and used to calibrate the bioluminescence intensity.

### Experimental guidelines and statistical methods

All experiments were carried out in strict accordance with the UKCCCR guidelines (Workman *et al*, 1998) and the German Law for Animal Protection of 1987. All procedures had also been reviewed by the responsible regional Ethics Committee. Kaplan–Meier statistics were applied to describe *in vivo* tumour growth characteristics and a log-rank analysis used to assess the significance of differences between the various treatment groups. All other analyses were carried out using the Wilcoxon test for unpaired samples. The significance level in all cases was set at *α*=5%.

## RESULTS

**Figure 2:**
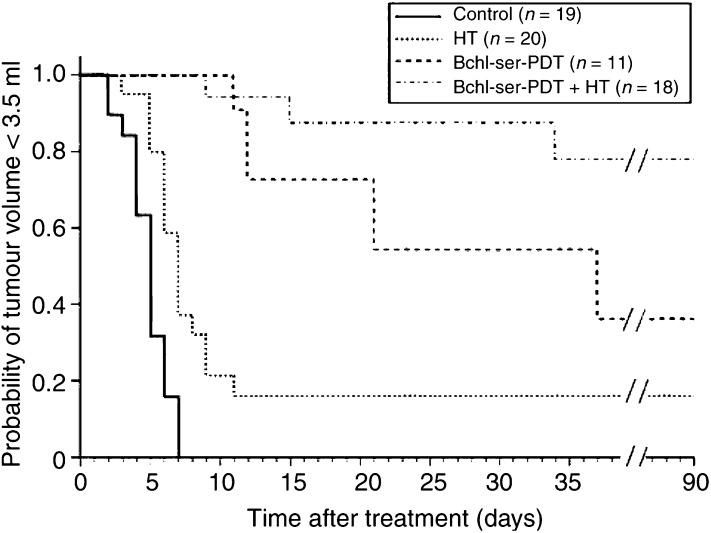
Kaplan–Meier analysis showing the probability of tumour volume being less than 3.5 ml as a function of time following sham-treatment (control), localised HT (43°C, 60 min), Bchl-ser-based photodynamic therapy (Bchl-ser-PDT; 20 mg kg^−1^ i.v.) or combined Bchl-ser-PDT+HT treatment. *n*=number of tumours treated.

All animals recovered rapidly following treatment, with no phototoxic effects on normal skin being recorded. Assessment of tumour volume for up to 90 days provided the basis for a Kaplan–Meier analysis, showing the probability of the tumour volume being less than 3.5 ml during the observation period (Figure 2

). Spontaneous cure was not seen in any of the sham-treated animals (control), with the target volume being reached approximately 7 days after treatment. Tumour treatment with localised 43°C HT resulted in a significant increase in the probability of tumours not reaching the target volume (16% on day 90) compared to the control group (*P*<0.001). Bchl-ser-PDT alone also effectively inhibited tumour growth with a probability of 36% on day 90 (*P*<0.001). The greatest inhibition of tumour growth was seen upon Bchl-ser-PDT+HT, with a probability of tumours not reaching the target volume of 78%, 90 days after treatment (*P*<0.001). Of the tumours which did not reach the target volume within the observation period, the majority showed no evidence of tumour presence on macroscopic examination. Only one tumour treated with Bchl-ser-PDT+HT showed slow tumour recurrence from day 30 after treatment onwards, which still had not reached the target volume by day 90. The differences between the Bchl-ser-PDT+HT and Bchl-ser-PDT groups, while not being significant upon log-rank analysis, do suggest a trend towards a greater antitumour effect with the combined therapy form (*P*=0.083). Considering this, the subsequent experiments were carried out to elucidate the impact of the combined therapy on tumour perfusion, oxygenation and metabolic microenvironment, in comparison to the effects of HT alone, since such effects may play a key role in the observed inhibition of tumour growth.

**Figure 3:**
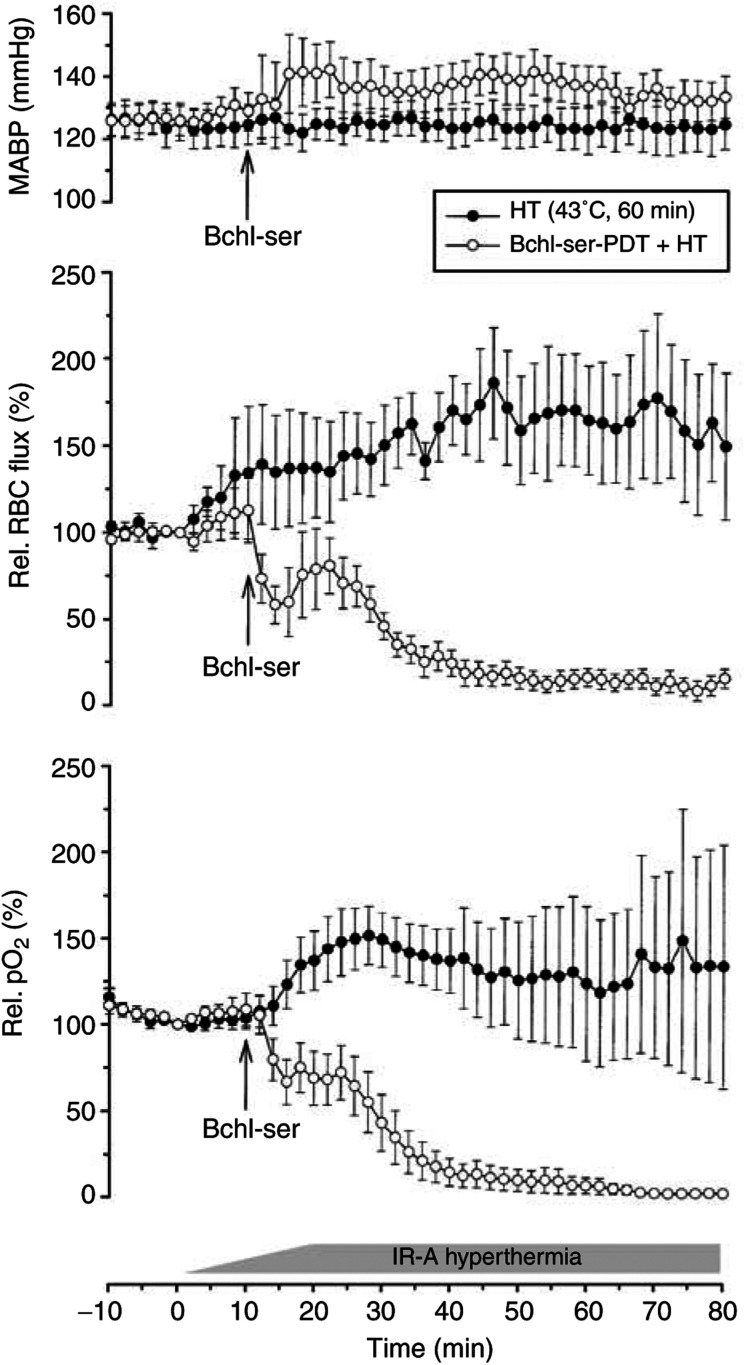
Mean arterial blood pressure (MABP; upper panel), relative RBC flux in tumour microvessels (centre panel) and tumour O_2_ tension (*p*O_2_, lower panel) as a function of time during localised HT (43°C, 60 min) or combined Bchl-ser-based photodynamic therapy and HT (Bchl-ser-PDT+HT) treatment. Each data point indicates the mean values (±s.e.m.) for a minimum of five tumours.

Changes in MABP, red blood cell flux (RBC flux) in tumour microvessels and tumour oxygenation over the whole observation period are shown in Figure 3

. Upon commencement of treatment with HT alone, a steady increase (up to 80% above initial values) in RBC flux was seen, with this elevation remaining until the end of the treatment period. Upon Bchl-ser-PDT+HT treatment, a characteristic biphasic change in RBC flux was seen. Within the first 15 min of treatment, RBC flux decreased to 50%. This decrease was followed by a transient, partial recovery, lasting approximately 10 min. Thereafter, a further steady decline in RBC flux was observed, reaching values approximately 10% of the initial flux. This low flux level endured throughout the remainder of the observation period (Figure 3, centre panel). Tumour oxygenation, as measured polarographically, showed similar changes to those seen for RBC flux. Upon HT treatment, an increase in *p*O_2_ was seen, which reached an apex at a level 50% above initial values approximately 30 min after the commencement of treatment. Thereafter, tumour *p*O_2_ remained elevated throughout the observation period. With Bchl-ser-PDT+HT treatment, a biphasic response was again seen, with an initial 30% decrease, followed by a transient plateau phase. Thereafter, a further steady decrease was seen reaching values approximating anoxic levels from 35 min onwards (Figure 3, lower panel). Since tumour red blood cell flux and oxygenation changes may be due to alterations in perfusion pressure, MABP was also monitored over the observation period (Figure 3, upper panel). During HT treatment, MABP remained constant. During Bchl-ser-PDT+HT treatment, a small increase in MABP (≈10 mmHg) was seen, which was maintained for the remainder of the observation period. This finding verifies that the decrease in tumour RBC flux and oxygenation seen upon Bchl-ser-PDT+HT treatment is not attributable to a decrease in perfusion pressure.

**Figure 4:**
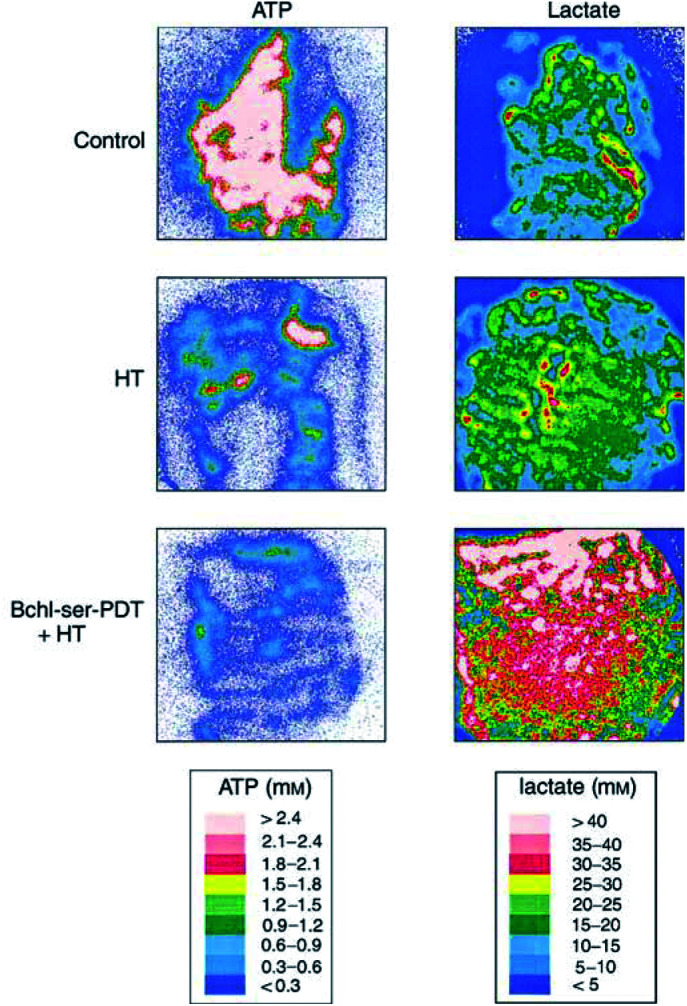
Examples of colour-coded distributions of ATP and lactate concentrations measured in cryosections of DS-sarcomas following sham treatment (control), localised hyperthermia (HT; 43°C, 60 min) or combined Bchl-ser-based photodynamic therapy and HT (Bchl-ser-PDT+HT) treatment.

Examples of colour-coded images of microregional ATP and lactate distributions in tumour sections obtained using single-photon imaging and bioluminescence techniques are shown in Figure 4

. Compared to images obtained in control tumours, treatment with HT caused a depletion of ATP levels, an effect that became even more pronounced upon Bchl-ser-PDT+HT treatment, where hardly any tumour areas with ATP concentrations >1 mM were seen. When the lactate distribution images were compared, HT appeared to induce a slight increase in lactate levels. Upon Bchl-ser-PDT+HT treatment, distinct and extensive increases in lactate concentrations were observed, with numerous regions showing concentrations >40 mM.

The treatment-induced changes in lactate and ATP concentrations seen using single-photon imaging and bioluminescence techniques were confirmed using methods assessing global metabolite concentrations (Table 1

**Table 1:** Global glucose, lactate and ATP concentrations in s.c. DS-sarcomas following sham-treatment (control), localised 43°C-HT or combined Bchl-ser-based photodynamic therapy and HT (Bchl-ser-PDT+HT)

**Treatment group**	***n***	**Glucose concentration (mM)**	**Lactate concentration (mM)**	**ATP concentration (mM)**
Control	9	2.03±0.29	12.68±2.01	0.99±0.24
HT	10	2.02±0.30	11.77±1.71	0.56±0.10
Bchl-ser-PDT+HT	9	2.51±0.39	22.87±2.42	0.27±0.11
			(*P*<0.01)	(*P*<0.05)

Values are presented as mean±s.e.m. *n*=number of tumours evaluated. Significance levels found upon testing differences between values obtained in control and treated tumours are given.

). Significant increases in lactate concentrations were only seen upon Bchl-ser-PDT+HT (*P*<0.01), where the concentration of lactate almost doubled upon treatment. ATP levels were reduced upon treatment, with Bchl-ser-PDT+HT causing the most pronounced reductions (*P*<0.05). Global glucose levels were also assessed, although no significant changes were evident.

## DISCUSSION

This paper describes for the first time the antitumour effect of combined Bchl-ser-PDT+HT, as compared to the action of Bchl-ser-PDT or HT alone. Additionally, data are presented on the influence of such a combined therapy on tumour perfusion, oxygenation and metabolic microenvironment. The effectiveness of such a combined approach is demonstrated by the high probability (78%) of tumours not reaching the target volume. Of the tumours observed up to day 90, the majority showed no evidence of tumour presence, thus underlining the therapeutic potential of PDT combined with HT.

A number of *in vitro* and *in vivo* studies have suggested a possible role for HT in combination with conventional PDT (Christensen *et al*, 1984; Mang and Dougherty, 1985; Chen *et al*, 1996; Rasch *et al*, 1996; Orenstein *et al*, 1999), although few of these studies have addressed the logistical difficulties of applying the two treatment components simultaneously, despite the fact that this may be desirable for application in the clinical setting. In the present study, an easily applicable technique was used to simultaneously apply Bchl-ser-PDT and HT using a single, nonlaser radiation source. The use of a nonlaser system is favourable, since the high purchase and maintenance costs of often complex laser systems are a major drawback to PDT achieving widespread clinical acceptance (Stringer, 1995).

The combination of an antivascular PDT treatment with localised HT, as applied in this study, is based on a number of considerations. Firstly, Bchl-ser is injected i.v. 10 min after commencement of HT, at a time when the tumour is still in the heating-up phase and has a temperature of approximately 40°C. At this point in time a mean increase in tumour RBF flux of ≈10% was observed, which could therefore be exploited to enhance the delivery of Bchl-ser to the tumour tissue. Secondly, the photodynamic process is known to be inherently temperature dependent (Gottfried and Kimel, 1991), and thus PDT in heated tissue should show an increased cytotoxic effect. Clinically, one obstacle to an effective hyperthermia treatment in human tumours is the inability to obtain a therapeutically relevant temperature elevation in the tumour tissue. This may be partly due to limitations of the heating equipment used, but also due to the relatively high perfusion rates that have been encountered in human tumours, leading to an unwanted dissipation of heat away from the malignancy (Vaupel and Kelleher, 1995). Thus, the combination of a vascular-based PDT with HT may facilitate and homogenise tissue heating by limiting the thermal wash-out. In other studies using vasoactive flow-reducing agents, the effects of thermal damage induced by HT were also shown to be augmented (for a review, see Prescott, 1995). More recently, the use of the vascular targeting agent combretastatin A-4 in combination with hyperthermia has further demonstrated the effectiveness of tumour heating in combination with a vascular targeting agent (Eikesdal *et al*, 2001). Thirdly, a reduction of tumour blood flow using vascular agents can cause an increase in tumour acidity (Griffin *et al*, 2003), which in turn can potentiate the effect of hyperthermia on tumour cells (Prescott, 1995). This phenomenon may also be important during Bchl-ser-PDT+HT treatment, since increased lactate concentrations were observed in tumour tissue during this treatment. While the primary effect of HT appears to be based on cellular and molecular mechanisms leading to tumour cell death, effects of HT on the microcirculation have been reported, with both increases and decreases in perfusion being found depending on the thermal dose applied and on the tumor model or – in the case of human tumours – on the pathology of the tumour investigated, dramatic variations being found between different regions of the same tumour (Kelleher *et al*, 1995). In the present study, HT alone resulted in a mean increase in tumour perfusion, suggesting that the Bchl-ser-PDT component is primarily responsible for the decrease in RBC flux, rapidly nullifying the 10% increase induced by HT treatment immediately prior to Bchl-ser administration. Of interest is also the biphasic course of the changes in RBC flux and oxygenation seen during Bchl-ser-PDT+HT. At present, the cause of this phenomenon is not clear, but could involve drug relocalisation as has been reported for other photosensitisers (MacDonald and Dougherty, 2001). Certainly, further investigations are warranted if the mechanisms underlying the exact antivascular effects of this therapy are to be understood. Based on the findings presented in this study, the therapeutic effectiveness of the Bchl-ser-PDT+HT treatment is mediated by a rapidly induced vascular stasis, which in turn can lead to the tumour cells being deprived of O_2_ and nutrients. In earlier studies in which the effects of Bchl-ser-PDT alone were monitored in tumour tissue, PDT-induced O_2_ consumption (Zilberstein *et al*, 1997) and changes indicative of vascular stasis (Zilberstein *et al*, 2001) were also found. While vascular damage has frequently been described as a consequence of PDT, it is not a primary target when PDT is applied in the clinical oncology setting, since, as with ionising radiation and some chemotherapy agents, the PDT effect is O_2_-dependent (Höckel and Vaupel, 2001) and thus, O_2_ deprivation due to vascular damage may reduce the treatment efficacy. In the case of Bchl-ser, however, a phototoxic effect has also been seen under hypoxic conditions (pO_2_ ≈2.5 mmHg) (Chen *et al*, 1998) and a model has been proposed in which the excitation of a Bchl-ser molecule coordinated with an oxygen molecule prior to illumination leads to dehydrogenation of the Bchl moiety, with the concomitant generation of hydroxyl radicals (Katz *et al*, 1998). In the present study, the *in vivo* effectiveness of Bchl-ser-PDT+HT, despite a rapid O_2_ depletion during treatment, would also suggest that O_2_ deprivation is not a limiting factor in this setting. This is further supported by a recent investigation of PDT-induced damage using a related bacteriochlorophyll derivative, Pd-bacteriopheophorbide as the photosensitiser in human prostate xenografts in mice (Koudinova *et al*, 2003). Here, the extent of PDT-induced damage was monitored by the immunohistochemical tracking of lipid peroxidation (LP). Lipid peroxidation products could be identified which were localised exclusively around blood vessels in the first hour after PDT, and were attributed to light-dependent ROS formation. The majority of the tumour however, showed evidence of LP 24–48 h after treatment in an apparently light-independent process. This damage is presumably associated with free radical-associated chain reactions initiated by PDT, which can take place regardless of blood stasis and lead to secondary tumour destruction due to a PDT-induced hypoxic state. Thus, this novel combined treatment may be particularly suitable for the treatment of hypoxic tumours or tumour regions, since both components appear able to exert a cytotoxic effect, even under conditions of O_2_ deficiency. This study has also demonstrated that significant metabolic changes can be observed directly after the combined therapy. The doubling of the lactate concentration and the ATP depletion correspond well with the changes seen in blood flow and oxygenation, and are suggestive of a switch within the tumour to glycolytic glucose turnover. Since PDT (and more so PDT in combination with HT) is a modality in which a number of treatment parameters related to the photosensitiser and light (drug dose, drug-light interval, light dose, etc) used need to be selected, studies have also been carried out in which magnetic resonance spectroscopy (MRS) techniques have been used to try to predict PDT outcome at the time of treatment. In one such study (Bremner *et al*, 1999), a clear relationship was seen between MRS measurements of energy metabolism immediately following PDT and the ultimate effect on the tumour in terms of regrowth delay. The data obtained in the present study would also support the concept that monitoring of ATP or lactate levels may provide information on the effectiveness of PDT-based treatments.

In conclusion, an effective tumour therapy in which Bchl-ser-PDT and HT have been combined and simultaneously applied has been described, and the results presented suggest that vascular collapse and flow stasis resulting in a deterioration of tumour oxygenation and a switch from oxidative to glycolytic glucose turnover are key elements in the tumour eradication seen with this novel approach.
